# Pregnant and Postpartum Women Requiring Intensive Care Treatment for COVID-19—First Data from the CRONOS-Registry

**DOI:** 10.3390/jcm11030701

**Published:** 2022-01-28

**Authors:** Magdalena Sitter, Ulrich Pecks, Mario Rüdiger, Sabine Friedrich, Sara Fill Malfertheiner, Alexander Hein, Josefine T. Königbauer, Karin Becke-Jakob, Janine Zöllkau, Babett Ramsauer, Katharina Rathberger, Constanza A. Pontones, Katrina Kraft, Patrick Meybohm, Christoph Härtel, Peter Kranke, CRONOS Network

**Affiliations:** 1 Department of Anaesthesiology, Intensive Care, Emergency and Pain Medicine, University Hospital Wuerzburg, 97080 Wuerzburg, Germany; sitter_m@ukw.de (M.S.); friedrich_s@ukw.de (S.F.); meybohm_p@ukw.de (P.M.); 2 Department of Obstetrics and Gynecology, University Hospital of Schleswig-Holstein, 24105 Kiel, Germany; ulrich.pecks@uksh.de; 3 Saxony Center for Feto-Neonatal Health, Technische Universität Dresden, Medizinische Fakultät, 01307 Dresden, Germany; mario.ruediger@ukdd.de; 4 Department of Gynecology and Obstetrics, Hospital St. Hedweig of the Order of St. John, University Medical Center Regensburg, 93049 Regensburg, Germany; sara.fillmalfertheiner@barmherzige-regensburg.de (S.F.M.); katharina.rathberger@barmherzige-regensburg.de (K.R.); 5 Department of Obstetrics and Gynaecology, Universitätsklinikum Erlangen, 91054 Erlangen, Germany; alexander.hein@uk-erlangen.de (A.H.); constanza.pontones@uk-erlangen.de (C.A.P.); 6 Department of Obstetrics and Gynecology, Vivantes Klinikum im Friedrichshain, 10249 Berlin, Germany; Josefine.koenigbauer@vivantes.de; 7 Department of Anesthesiology and Intensive Care, Klinik Hallerwiese-Cnopfsche Kinderklinik, 90419 Nurnberg, Germany; Karin.becke-jakob@diakoneo.de; 8 Department of Obstetrics, University Hospital Jena, 07747 Jena, Germany; Janine.zoellkau@med.uni-jena.de; 9 Department of Obstetrics, Vivantes Klinikum Neukölln, 12351 Berlin, Germany; babett.ramsauer@vivantes.de; 10 Department of Obstetrics and Gynecology, München Klinik Harlaching, 81545 Munich, Germany; Katrina.kraft@outlook.de; 11 Department of Paediatrics, University Hospital Wuerzburg, 97080 Wuerzburg, Germany; haertel_c1@ukw.de

**Keywords:** maternal critical care, COVID-19, ARDS, SARS-CoV-2, pregnancy, obstetrics

## Abstract

(1) Background: Data on coronavirus 2 infection during pregnancy vary. We aimed to describe maternal characteristics and clinical presentation of SARS-CoV-2 positive women requiring intensive care treatment for COVID-19 during pregnancy and postpartum period based on data of a comprehensive German surveillance system in obstetric patients. (2) Methods: Data from COVID-19 Related Obstetric and Neonatal Outcome Study (CRONOS), a prospective multicenter registry for SARS-CoV-2 positive pregnant women, was analyzed with respect to ICU treatment. All women requiring intensive care treatment for COVID-19 were included and compared regarding maternal characteristics, course of disease, as well as maternal and neonatal outcomes. (3) Results: Of 2650 cases in CRONOS, 101 women (4%) had a documented ICU stay. Median maternal age was 33 (IQR, 30–36) years. COVID-19 was diagnosed at a median gestational age of 33 (IQR, 28–35) weeks. As the most invasive form of COVID-19 treatment interventions, patients received either continuous monitoring of vital signs without further treatment requirement (*n* = 6), insufflation of oxygen (*n* = 30), non-invasive ventilation (*n* = 22), invasive ventilation (*n* = 28), or escalation to extracorporeal membrane oxygenation (*n* = 15). No significant clinical differences were identified between patients receiving different forms of ventilatory support for COVID-19. Prevalence of preterm delivery was significantly higher in women receiving invasive respiratory treatments. Four women died of COVID-19 and six fetuses were stillborn. (4) Conclusions: Our cohort shows that progression of COVID-19 is rare in pregnant and postpartum women treated in the ICU. Preterm birth rate is high and COVID-19 requiring respiratory support increases the risk of poor maternal and neonatal outcome.

## 1. Introduction

The current pandemic caused by the severe acute respiratory syndrome coronavirus 2 (SARS-CoV-2) is affecting populations and health care systems worldwide. As of today, over 250 million people have been infected by the virus and it has caused over 5 million deaths [1]. In Germany, over 5 million infections have been reported and over 100,000 patients have died from or with coronavirus disease 2019 (COVID-19) [2]. COVID-19 has led to a very high demand for critical care beds, resulting in the exhaustion of intensive care capacity in many health systems [3].

SARS-CoV-2 is a coronavirus classified in the same subgroup as the severe acute respiratory syndrome coronavirus 1 and the Middle East respiratory syndrome coronavirus. Both can cause a variety of respiratory illnesses ranging in severity from a common cold to severe pneumonia, inflammatory response, acute lung injury, and death [4,5]. Research on COVID-19 revealed that SARS CoV-2 does not only affect the respiratory tract but can also lead to endothelial inflammation, cardiomyopathy, multi-organ dysfunction, neurological syndromes, and hypercoagulability [6].

Due to changes in the respiratory physiology during pregnancy, including a decrease of residual volume and increase of respiratory resistance, as well as other physiological changes such as immunological modulations towards a tolerogenic state and elevated cardiac strain, pregnant women are considered to be at increased risk for severe illness with regard to respiratory infections [7,8]. Pregnant women infected with SARS-CoV-1 or influenza (e.g., H1N1) have a reported higher morbidity and mortality rate as a result of associated pneumonia and acute respiratory distress syndrome (ARDS) [9]. Based on these reports, the SARS-CoV-2 pandemic raised concerns about similar risks for pregnant women with COVID-19. Although data for this vulnerable subgroup have been published, many questions remain concerning risk factors, clinical features, and treatment approaches for pregnant women and parturients requiring intensive care treatment for COVID-19, as well as decisions regarding the timing and mode of delivery.

To support clinicians treating pregnant and postpartum patients with COVID-19, the German Society for Perinatal Medicine initiated a registry study in April 2020: ‘COVID-19 Related Obstetric and Neonatal Outcome Study’ (CRONOS) [10]. We evaluated women who were admitted to intensive care units (ICU) in Germany (and Linz, Austria) following SARS CoV-2 infection during pregnancy.

This report aims to describe the obstetric and general physical short-term outcome of SARS-CoV-2 positive women receiving ICU treatment for COVID-19 during pregnancy and postpartum. Identification of obstetric and COVID-19 specific risk factors for disease progression, outcome, and therapeutic strategies in the management of COVID-19 in pregnancy may provide further insights into management strategies.

## 2. Materials and Methods

### 2.1. CRONOS-Registry

COVID-19 Related Obstetric and Neonatal Outcome Study (CRONOS) is an ongoing prospective multicenter registry study for SARS-CoV-2 positive pregnant women, initiated on 3 April 2020 by the German Society of Perinatal Medicine. Ethics approval was obtained (University Hospital Schleswig-Holstein in Kiel, file number D 451/20). Waiver for informed consent was approved if identifiable protected health information could be omitted. All hospitals were advised to obtain informed consent. The study was registered in the German Registry for Clinical Studies (DRKS00021208). An electronic case report form (eCRF) was developed using the cloud-based electronic data capture platform of the service provider Castor EDC [11]. By 24 August 2021, obstetricians and neonatologists from 157 German hospitals and from Kepler University Hospital Linz, Austria, confirmed to participate.

Women with confirmed SARS-CoV-2 infection (RT-PCR or serological tests) at any time during pregnancy were included. In addition to COVID-19-specific symptoms and treatments, information related to pregnancy and birth as well as neonatal outcomes were collected. The data were entered by each hospital. The collected data were extracted from clinical documents and individual maternity logs. The registry included patients’ baseline data, medical history, as well as COVID-19 and treatment related aspects. The further course of the pregnancy, the obstetric outcome, the postpartum period, and the neonatal outcome were reported up to six weeks postpartum.

### 2.2. Patient Selection

All cases with confirmed SARS-CoV-2 infection at any time during pregnancy at any of the participating centers between 3 April 2020 and 24 August 2021 as entered into the CRONOS registry were reviewed. Cases with documented ICU admission were used for the current analysis.

### 2.3. Additional Data Collection for ICU Population

**Figure 1:**
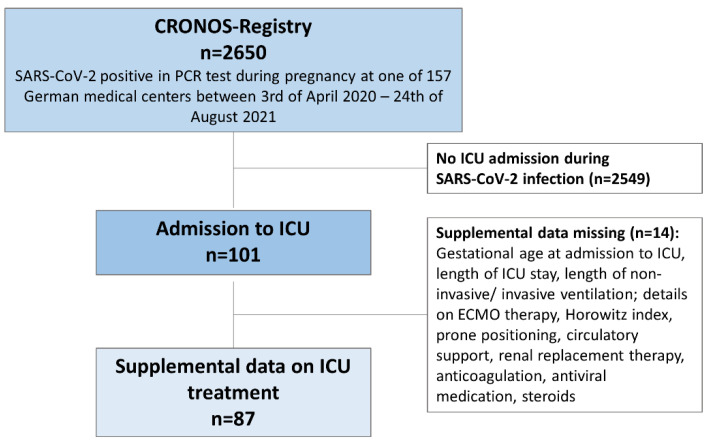
Study flow of patient selection and supplemental data collection. Patients without completed supplemental data collection were not excluded from this study. Eighty-seven patients were included for the analysis performed on supplemental data.

Supplemental questionnaires to collect information describing the intensive care treatment were distributed to centers reporting ICU admissions. The questionnaire was updated after review of the first 25 cases and after review and analysis of 50 cases. Data were validated weekly by members of the CRONOS organizational team and centers were contacted if discrepancies were found. Figure 1 displays patient selection and additional data collection for the ICU population.

As a surrogate marker of disease severity, patients were categorized based on the most invasive respiratory support required during the course of the treatment. Lowest to highest severity was defined as follows: continuous monitoring and requirement of oxygen insufflation (e.g., by nasal prongs) representing moderate disease score 4 and 5 in accordance with the WHO clinical progression scale [12]; non-invasive ventilation (NIV, including high-flow nasal oxygen therapy), invasive ventilation through an endotracheal tube, and extracorporeal membrane oxygenation (ECMO) representing severe disease (score 6–9, WHO clinical progression scale).

### 2.4. Statistical Analyses

Data are reported as medians and interquartile range (IQR, 25–75%) (continuous data) or absolute and percentages (categorical data). Normality of the data could not be assumed after evaluation with graphical methods. Two-sided statistical significance was set at *p* < 0.05. Kruskal-Wallis-Test was applied for metric data. If statistical significance was found in intergroup analysis, post-hoc analysis was performed using Wilcox rank sum test. We performed Chi-square test of independence to examine differences in maternal age, body mass index (BMI) before pregnancy, and frequencies of pre-existing medical conditions between the five varying forms of respiratory support. If statistical significance was found, logistic regression was performed.

Descriptive data analysis, statistical analysis, and visual presentation was performed with R (R Core Team, 2020), using packages including ggplot2 (Wickham, 2009).

## 3. Results

### 3.1. Patient Population

From 3 April 2020 to 24 August 2021, a total of 2650 patients with a diagnosed SARS-CoV-2 infection during pregnancy were included in the CRONOS-registry. We identified and analyzed 101 SARS-CoV-2 positive women, who received intensive care treatment for COVID-19 during pregnancy and postpartum from 50 different German hospitals. SARS-CoV-2 infection was confirmed by a polymerase chain reaction (PCR) test. Supplemental data collection was completed for a subgroup of 87 patients. In 14 cases the questionnaire for supplemental data collection was not completed, so that only eCRF data could be analyzed. Figure 1 describes patient selection and supplemental data collection.

Eighty-four patients required ICU treatment solely due to COVID-19 with respiratory symptoms including pneumonia and ARDS. Seventeen required ICU treatment due to combined complications of pregnancy and COVID-19.

### 3.2. Maternal Characteristics

Patients had a median age of 33 (IQR, 30–36) years. More than two thirds of all patients were diagnosed with SARS-CoV-2 during the third trimester (73%), 26 patients during the second, and one patient during the first trimester of pregnancy. An overview of maternal characteristics is shown in Table 1. Table 2 displays the medical history, as well as clinical findings at SARS-CoV-2 diagnosis.

### 3.3. ICU Treatment—Stage of Pregnancy

**Figure 2:**
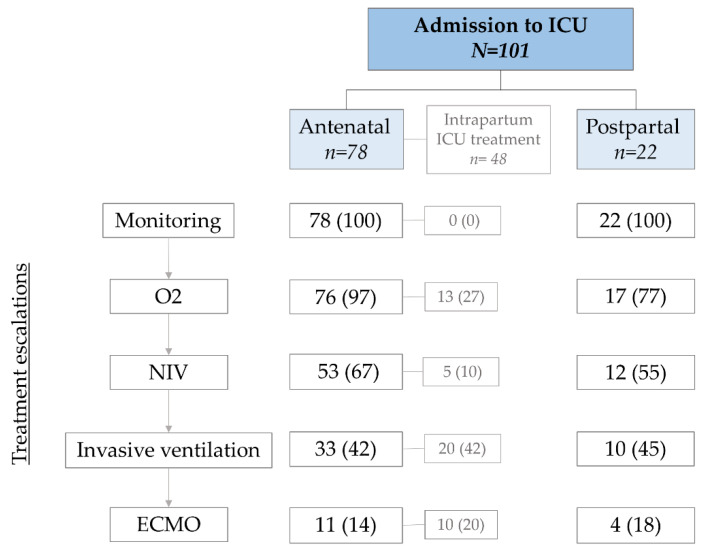
Admission to ICU and treatment escalations at different stages of pregnancy. This figure is based on the clinical assumption that treatments are gradually escalated until highest treatment required is reached; antenatal and postpartum: numbers presented as flow chart; intrapartum: total number of patients receiving ICU treatment during childbirth; intensive care unit (ICU), insufflation of oxygen (O^2^), non-invasive ventilation (NIV), invasive ventilation, and extracorporeal membrane oxygenation (ECMO).

As our cohort includes pregnant and peripartumwomen, admission to ICU and ICU treatments took place at different time points during pregnancy or the postpartum period. In Figure 2 we display the distribution of patients according to the time point of admission to ICU, as well as the treatment escalations under the assumption of a clinically based step by step approach toward invasive treatments.

### 3.4. ICU Treatment—Respiratory Treatment

**Figure 3:**
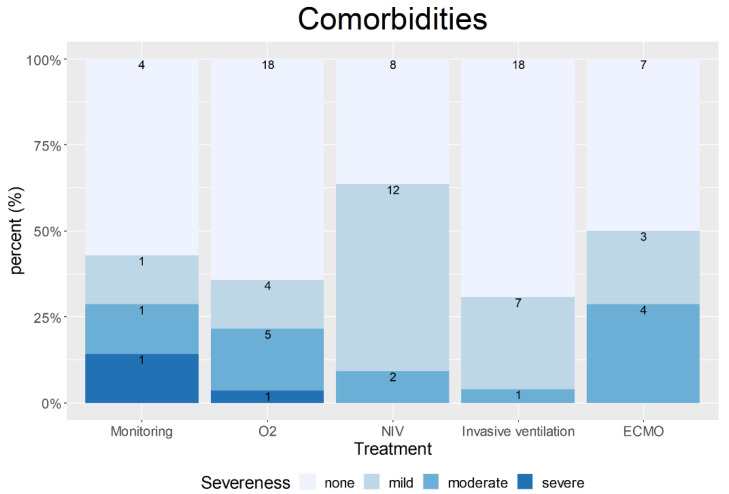
Distribution of none, mild, moderate or severe pre-existing conditions between treatments. Pre-existing comorbidities were scored by the treating physician. Mild disease was defined as pre-existing illnesses without any medical impairments, moderate disease as disease with mild dysfunctions and need for regular medication intake, and severe disease as requiring regular medical attention. Treatments: monitoring; insufflation of oxygen (O^2^); non-invasive ventilation (NIV); invasive ventilation; and extracorporeal membrane oxygenation (ECMO).

Overall, ICU admission occurred within one week after SARS-CoV-2 diagnosis (range, 0 to 3 weeks). Median ICU length of stay was 9 days (IQR, 4–19). Differences between forms of respiratory support regarding patient characteristics and treatment modalities are shown in Table 3. Distribution of maternal age, body mass index (BMI) at admission, and gestational age at diagnosis did not vary significantly following treatment stratification as a measure of disease severity. Figure 3 displays the distribution of comorbidities according to the treatment requirement. No statistically significant differences were found between treatments and the frequencies of pre-existing medical conditions; in fact, the two women with severe pre-existing medical conditions in this cohort required the two lowest treatment escalations for COVID-19 (continuous monitoring and insufflation of oxygen).

**Figure 4:**
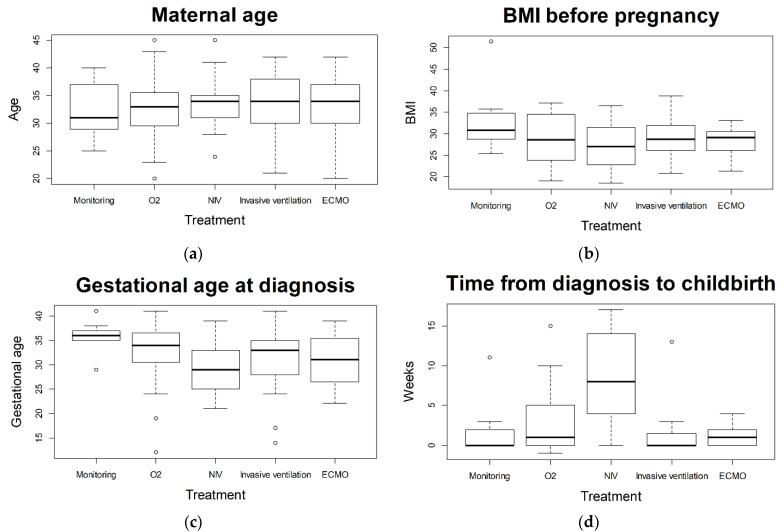
Comparison of (**a**) maternal age; (**b**) BMI before pregnancy; (**c**) gestational age at diagnosis; and (**d**) time from diagnosis to childbirth. Values were compared between treatments: monitoring, insufflation of oxygen (O^2^), non-invasive ventilation (NIV), invasive ventilation, extracorporeal membrane oxygenation (ECMO), and body mass index (BMI). Statistically significant differences were observed for “Gestational age at diagnosis” (*p* = 0.02) and “Time from diagnosis to childbirth” (*p* = 0.00034).

Figure 4 displays differences of various maternal characteristics and aspects of pregnancy between treatments applied for COVID-19. Statistically significant differences were found for the following aspects: “gestational age at diagnosis” (*p* = 0.026, post-hoc test: O^2^ vs. NIV *p* = 0.079); “gestational age at childbirth” (*p* = 0.000005 post-hoc test shows statistically significant differences between the non-invasive treatments (monitoring, O^2^, NIV) and the invasive treatments (ITN, ECMO)); “time from diagnosis to childbirth” (*p* = 0.00034, post-hoc test shows statistically significant differences between NIV and the treatments O^2^, ITN, and ECMO).

Six patients were admitted to the ICU for continuous monitoring without the need of any further ICU treatment. One woman was admitted to the ICU for closer monitoring due to SARS-CoV-2 infection and maternal exhaustion following spontaneous delivery without the need of any specific treatment for COVID-19. Two women were monitored in the ICU for hemolysis elevated liver enzymes and low platelets (HELLP) syndrome concomitant to SARS-CoV-2 infection. One woman was admitted to the ICU for close monitoring after postpartum hemorrhage due to uterine atony. Two women were admitted to ICU due to metabolic acidosis: in one case the metabolic acidosis was related to nausea and vomiting, whilst the other case was related to dysgeusia followed by food abstinence.

Thirty patients required supplemental oxygen as the highest level of treatment. Ten out of 23 patients with antepartum ICU stay recovered from COVID-19 prior to childbirth.

A total of 22 patients required NIV (non-invasive ventilation or high flow via nasal cannula (HFNC) alone (*n* = 13 (59%)) or a combination (*n* = 9 (41%)). In this cohort of patients, fifteen (*n* = 22 (68%)) women recovered from COVID-19 before childbirth. Median time from infection to childbirth was 8 weeks (IQR, 4–14). This resulted in a statistically significant pregnancy prolongation in comparison to the other treatments (*p* < 0.00034). One (*n* = 22 (5%)) patient was admitted to the ICU requiring NIV therapy within 7 days after delivery.

Invasive ventilation was required in a total of 28 patients. Three women (11%) recovered from COVID-19 prior to childbirth and eleven (39%) patients after delivery; further information on the recovery was unknown in nine (32%) cases. Twenty-five (89%) women were delivered by caesarean section, two (7%) gave birth spontaneously, and one (4%) recovered prenatally but was lost to follow up.

Treatment escalation to ECMO therapy was required in 15 patients. One woman recovered. The pregnancy was medically terminated following severe fetal brain injury resulting from maternal hypoxia. Fourteen (87%) women required veno-venous ECMO therapy, in one case veno-arterial cannulation was necessary which was deescalated to veno-venous ECMO after five days. This patient received a double-lung transplantation two months after SARS-CoV-2 diagnosis. Median time of ECMO therapy was 25 (IQR, 12–41) days and first cannulation was performed in the first week after ICU admission (range; 1–14 days). One patient was cannulated three times before successful weaning from ECMO support. Five (33%) women recovered after childbirth and two (13%) women died.

A total of 69 patients received anticoagulants. Eleven thromboembolic events were reported (pulmonary embolism (*n* = 4) catheter associated thrombosis (*n* = 1), hypercoagulability during ECMO therapy with oxygenator clotting (*n* = 1), 5 not further specified). Eight patients had major bleeding: one cerebral hemorrhage while receiving ECMO therapy, one naso-pharyngeal bleeding while receiving ECMO therapy, two ECMO associated coagulopathies with bleeding at the cannula puncture site, and four peripartal hemorrhages due to uterine atony.

### 3.5. Obstetric Outcome

Time between positive SARS-CoV-2 testing and childbirth was between 0 and 17 weeks (information available in 83 patients). Of all patients, distribution of mode of delivery was as follows: 17 spontaneous deliveries, 1 operative assisted vaginal delivery, 52 primary caesarean sections, 11 secondary caesarean sections after onset of labor, and 2 emergency caesarean sections. In 18 pregnancies mode of delivery was not reported due to an ongoing pregnancy or the patients were lost to follow-up after prenatal recovery.

In 38 cases, iatrogenic delivery was deemed necessary to optimize maternal treatment for COVID-19 (e.g., prone positioning). Within this cohort, in 23 patients the clinical status deteriorated significantly post-delivery, with seven (*n* = 23, 30%) patients requiring ECMO therapy. Three (*n* = 23, 13%) women delivered spontaneously, 20 (*n* = 23, 87%) were delivered through caesarean section (emergency *n* = 1, primary *n* = 16, secondary *n* = 3). Seven (30%) patients had general anesthesia with invasive ventilation during delivery and eight (35%) received neuraxial anesthesia.

### 3.6. Neonatal Outcome

Forty-eight infants were born premature (i.e., delivery before completion of 37 gestational weeks). We observed significantly higher rates of premature delivery in patients with intensified care of COVID-19 (*p*-value 0.0004). Logistic regression did not show a statistically significant difference in premature birth rates between the five treatments. Grouping for non-invasive (monitoring, insufflation of oxygen, and non-invasive ventilation) and invasive treatment forms (invasive ventilation and ECMO) as well as antenatal admission to ICU revealed significantly higher odds of premature birth for invasive respiratory support (*p*-value < 0.001, OR 38.5 (95% CI 8.92–275.91)). Forty-nine infants required admission to the neonatal ICU (e.g., prematurity-related problems, respiratory maladaptation). A particular finding for one preterm infant (30 weeks of gestation) was intraventricular hemorrhage I°.

Six fetuses (9%) were stillborn: two due to abortion before 22 weeks, three fetal deaths at ≥28 weeks of gestation, and one medically indicated termination of pregnancy due to severe fetal brain damage following maternal hypoxia before admission to the hospital. No postnatal death of a neonate was registered. Swabs were taken from 46 of 78 live born neonates for PCR testing for SARS-CoV-2 RNA at birth: five neonates (11%) tested positive. One infant tested positive for SARS-CoV-2 at the age of 12 days. Fourteen neonates underwent antibody testing, of which three (21%) neonates were positive for SARS-CoV-2 antibodies. The mothers of these three neonates required antenatal intensive care treatment (one ECMO, 2 NIV). Table 4 displays maternal and neonatal outcomes for each treatment for COVID-19.

### 3.7. Follow-Up of Mother–Infant Dyads

Follow up visits were completed for 47 women at a median of 8 (IQR; 4–9) weeks after childbirth. No specific findings were reported in 35 cases. The other 12 woman reported a variety of post-COVID symptoms, i.e., impairment of daily life activities and problems due to thromboembolic events, tachycardia, and post-traumatic stress disorder. There were no significant findings in the follow-up of surviving infants.

## 4. Discussion

Data of pregnant women suffering from severe COVID-19 are sparse and knowledge is limited. Due to differences in health care systems, a regional analysis may be helpful. We analyzed 101 women requiring ICU treatment subsequent to SARS-CoV-2 infection during pregnancy based on the German CRONOS registry.

We found that the number of pregnant and postpartum women requiring intensive care treatment for COVID-19 is relatively low (here 101 out of 2650; 4%). However, in cases of severe disease progression, intensive care and escalation up to ECMO therapy may be required in spite of the low prevalence of pre-existing comorbidities. Maternal characteristics (such as maternal age, BMI, or pre-existing conditions) did not vary significantly following treatment stratification as a measure of disease severity. There were statistically significant differences with regard to gestational age at childbirth and rate of preterm delivery between treatment escalations. Although logistic regression could not identify a statistically significant difference between treatment requirements, grouping for non-invasive and invasive treatments revealed a significantly higher risk for premature birth. Admission to the ICU was associated with an elevated risk for a poor maternal and fetal outcome. Maternal mortality rate is higher for those requiring ICU treatment (5% in our cohort) than for pregnant women with COVID-19 (0.7%) or the comparison groups (0.2%), described by Allotey et al. [13]. Rate of stillbirth (6%) is also higher in ICU admitted patients compared to all pregnant women with COVID-19 (0.9%) and comparison groups (0.5%) [13]. Nevertheless, patients with severe COVID-19 not requiring invasive ventilation (non-invasive therapy as highest level of therapy) seem to benefit from treatment and show a high rate of recovery before childbirth without preterm labor.

Previous studies and reviews have shown that most affected women with COVID-19 during pregnancy show no or mild symptoms [14]. Clinical presentation and symptoms seem to be similar as for non-pregnant adults [13,15,16]. Our analysis yields similar conclusions, with cough, dyspnea, and malaise being the most frequently reported symptoms [13,17].

Comparing symptomatic women to non-pregnant women with similar risk profiles (age, pre-existing conditions, ethnicity, etc.), the course of COVID-19 has been shown to be significantly more severe in pregnant women. Corresponding analyses of 409,462 symptomatic, laboratory-confirmed SARS-CoV-2 infected women of reproductive age (15–44 years) showed a significantly increased risk in pregnant women for the need for intensive care (aRR 3.0; 95% CI 2.6–3.4) or death (aRR 1.7; 95% CI 1.2–2.4) as compared to non-pregnant women [18].

In comparison to pregnant women negative for SARS-CoV-2, an increased risk for maternal mortality has been described [19,20]. Furthermore, higher rates of in-hospital maternal death, preeclampsia, and thrombotic events have been reported [17,19].

In the abovementioned analyses, neither ICU treatment nor ICU treated patients were analyzed in detail. Easter et al. compared critically ill pregnant women with COVID-19 to non-pregnant patients, but a detailed analysis of differences in treatment modalities or severity of disease amongst ICU patients was not reported [21]. Describing the subgroup of SARS-CoV-2 positive pregnant women requiring ICU treatment according to severity status, as applied in this analysis, allows a more differentiated look at the different course of disease for SARS-CoV-2 infection during pregnancy leading to ICU admission.

With 101 of 2650 registered pregnant patients, the observed 4% ICU admission rate is similar to previously reported rates (3–10%) [16,17,18]. Although pregnant patients who tested positive for SARS-CoV-2 had high rates of preterm delivery, caesarean section, and perinatal death [16,20,21], Easter et al. raised the question of whether or not delivery is required for non-obstetric indications among critically ill pregnant women [21]. In our cohort we observed a high number of iatrogenic deliveries with a subsequent high rate of preterm birth, particularly among invasively treated patients. A noteworthy number of patients required NIV therapy (15 out of 101) and invasive ventilation (2 out of 101) and recovered prior to childbirth without the need for premature delivery due to COVID-19. We therefore suggest that a delivery is not necessarily required among ICU admitted pregnant women for non-obstetric reasons in otherwise stable patients. If the mother’s general condition deteriorates, the indication of delivery should be balanced between benefits for the mother (prone position, ECMO) and risks of prematurity for the infant.

Some authors postulated an increase of maternal morbidity with progression of pregnancy [22]. Of our ICU admitted patients, 71% tested positive for SARS-CoV-2 in the third trimester. Soheili et al. reported a higher risk of COVID-19 in the third trimester compared to the first and second trimester [23]. We found a statistically significant correlation between gestational age at diagnosis and severity of disease when comparing the group receiving insufflation of oxygen as highest form of treatment (later in gestational age) and the non-invasive ventilated patients (earlier in gestational age). The trend we observed showed a correlation between earlier week of gestational age at diagnosis and more severe course of disease (see Figure 4c).

Limitations of our study include population-based analysis with description of cases of SARS-CoV-2 positive women requiring ICU treatment for COVID-19 during pregnancy and postpartum. The small number of patients with various disease severities and the reporting bias/missing datasets within the registry are further limitations.

The sample population of 101 patients can be assumed to be an accurate representation of severe COVID-19 cases in the German obstetric population. The prospective CRONOS registry captures obstetric cases of more than 150 centers, representing 37% of births per year (ranging from primary care facilities to academic centers) throughout Germany and Linz, Austria. The diversity of data permits a closer look at the characteristics of this obstetric cohort, at the heterogeneity of COVID-19 related ICU treatment options and disease severities, and the sequelae of COVID-19 in pregnant women.

## 5. Conclusions

The data of this cohort show that ICU admission due to COVID-19 during pregnancy may happen without identifiable risk factors. Although the risk of poor maternal or neonatal outcome increases when ICU admission is required, adequate respiratory support adapted to the clinical status and interdisciplinary management of critical cases may not only lead to maternal recovery, but also an improvement in neonatal outcomes. Further research investigating the differences in treatment of severe and critical COVID-19 is necessary.

## Figures and Tables

**Table 1:** Maternal characteristics of SARS-CoV-2 positive pregnant and postpartum women requiring intensive care treatment for COVID-19.

	N = 101
Maternal age at SARS-CoV-2 infection (years)	33 (30–36)
<20 years	2 (2)
20–34 years	69 (68)
≥35 years	30 (30)
Multiple pregnancy	4 (4)
Gestational age	33 (28–35)
Trimester enrolled	
1st trimester	1 (1)
2nd trimester	26 (26)
3rd trimester	74 (73)
Ethnic origin	
Middle East	34 (34)
Northern Europe	30 (30)
Africa (others)	11 (11)
Eastern Europe	9 (9)
South East Asia	8 (8)
Southern Europe	2 (2)
Northern Africa	1 (1)
Open/Unknown	6 (6)
Australia, New Zealand	0 (0)
North America	0 (0)
Baseline data	
Body height (cm)	164 (160–166)
Body weight before pregnancy (kg)	77 (65–80)
Body weight at inclusion (kg)	85 (72–98)
BMI before pregnancy (kg/m^2^)	31.2 (26.3–33.0)
Underweight (<18.5)	1 (1)
Normal weight (18.5–25.0)	18 (18)
Overweight (25.0–30.0)	27 (27)
Obese (>30.0)	26 (26)
Unknown	29 (29)
BMI at inclusion (kg/mg^2^)	32 (28.6–34.2)
History of smoking before pregnancy	1 (1)
Smoking during pregnancy	1 (1)
Passive smoking	5 (5)

N (*n*%), median (IQR); severe acute respiratory syndrome coronavirus type 2 (SARS-CoV-2), body mass index (BMI).

**Table 2:** Pre-existing medical conditions, concomitant medication as well as symptoms, and laboratory and radiological findings at SARS-CoV-2 diagnosis.

Medical History	N = 101
Pre-existing illnesses ^a^	
None	54 (53)
Mild	27 (27)
Moderate	13 (13)
Severe	2 (2)
Pre-existing conditions	
Hypertension	3 (3)
Diabetes	
Diabetes mellitus Type I	1 (1)
Diabetes mellitus Type II	3 (3)
Gestational Diabetes	16 (16)
Thyroid Disease	
Hypothyroidism	5 (5)
Asthma	4 (4)
Hepatitis B	3 (3)
Others	5 (5)
Concomitant Medication	
None	63 (62)
Antibiotics	6 (6)
Acetylsalicylic Acid	3 (3)
Other NSAIDs	2 (2)
Immunosuppressive Drugs	1 (1)
Antihypertensive Drugs	5 (5)
Asthma Medication	1 (1)
Antidiabetic Drugs	10 (10)
Heparin derivative	11 (11)
Important Others	3 (3)
COVID-19	
Symptoms ^b^	*n* = 99 (98)
Number of symptoms per patient	6 (±3)
Cough	79 (78)
Dyspnea	84 (83)
Malaise	77 (76)
Fever	70 (69)
Fatigue	57 (56)
Clinical examination—diagnostic Imaging	
Imaging	*n* = 45 (45)
Ultrasound examination of the lungs	5 (11)
Thoracic X-ray	31 (69)
Thoracic CAT-Scan	14 (31)
Thoracic MRI	2 (4)
Radiological Findings typical for COVID-19	41 (91)

N (*n*%), mean (±SD), median (IQR); severe acute respiratory syndrome coronavirus type 2 (SARS-CoV-2), coronavirus disease 2019 (COVID-19), nonsteroidal anti-inflammatory drug (NSAID), computed tomography scan (CT-Scan), magnetic resonance imaging (MRI). ^a^ Pre-existing comorbidities were scored by the treating physician and categorized in the registry. Mild disease was defined as pre-existing illnesses without any medical impairments, moderate disease as disease with mild dysfunctions and need for regular medication intake, and severe disease as requiring regular medical attention. ^b^ Five most frequently reported symptoms displayed.

**Table 3:** Patients’ characteristics, details, and features applied for each treatment for COVID-19.

	Treatment for COVID-19					All N = 101 ^a^
	Monitoring *n* = 6	O^2^ *n* = 30	NIV *n* = 22	ITN *n* = 28	ECMO *n* = 15	
**General Information**						
Maternal age (years), mean (±SD)	30 (29–34)	33 (30–36)	34 (31–35)	34 (30–38)	34 (30–37)	34 (30–36)
Booking BMI (kg/m^2^)	31 (29–35)	29 (24–34)	27 (23–31)	29 (25–32)	29 (26–30)	31 (26–33)
Gestational age at diagnosis (weeks), median (IQR)	36 (35–36)	35 (31–37)	29 (25–33)	33 (28–35)	31 (27–36)	33 (28–35)
Gestational age at childbirth (weeks) ^b^, median (IQR)	37 (36–40)	38 (36–39)	39 (37–41)	33 (29–36)	33 (31–36)	36 (33–39)
Time between diagnosis and childbirth (weeks) ^c^, median (IQR)	0 (0–2)	1 (0–5)	8 (4–14) ^b^	0 (0–2)	1 (0–2)	3 (0–4)
Length of ICU stay (days), median (IQR)	1 (1–2)	4 (2–5)	7 (5–9)	20 (11–28)	38 (16–71)	9 (4–19)
Duration of treatment (days), median (IQR)	-	-	4 (3–6)	10 (3–16)	25 (12–41)	-
Horowitz Index (mmHg), median (IQR)	-	-	180 (136–233)	85 (68–176)	60 (50–67)	120 (67–184)
Prone position ^a^	-	-	4 (18)	13 (57)	9 (60)	26 (30)
Circulatory support ^a^	0 (0)	0 (0)	4 (18)	16 (70)	13 (87)	33 (41)
Renal Replacement Therapy ^a^	0 (0)	0 (0)	0 (0)	2 (9)	3 (20)	5 (6)
Thromboembolic event	1 (17)	1 (3)	0 (0)	3 (13)	6 (40)	11 (13)
Anticoagulation ^a^						
Prophylactic	1 (17)	13 (62)	14 (64)	12 (52)	6 (40)	46 (53)
Therapeutic	0 (0)	7 (33)	3 (14)	7 (30)	3 (20)	20 (23)
Antiviral medication ^a^	0 (0)	2 (10)	3 (14)	5 (22)	5 (33)	15 (17)
Steroids ^a^	0 (0)	8 (38)	14 (64)	17 (74)	7 (47)	46 (53)

Numbers are presented as frequency (%) if not otherwise specified. Coronavirus disease 2019 (COVID-19), insufflation of oxygen (O^2^), non-invasive ventilation (NIV), intubation for invasive ventilation (ITN), extracorporeal membrane oxygenation (ECMO), intensive care unit (ICU), and interquartile range (IQR). ^a^ If information is taken out of the questionnaire (subgroup analysis): total *n* = 87, monitor N = 6, O^2^ N = 21, NIV N = 22, ITN N = 23, ECMO N = 15. ^b^ *p*-value = 0.000005, ^c^ *p*-value = 0.00034.

**Table 4:** Maternal and neonatal outcomes for each treatment for COVID-19.

	Treatment for COVID-19					All N = 101 ^a^
	Monitoring n = 6	O^2^ n = 30	NIV n = 22	ITN n = 28	ECMO n = 15	
Maternal outcome						
Recovery	5 (83)	20 (67)	16 (73)	19 (68)	6 (40)	66 (65)
Death	0 (0)	0 (0)	0 (0)	1 (4)	3 (20)	5 (5)
Unknown/open	1 (17)	10 (33)	6 (27)	8 (35)	7 (47)	32 (32)
Neonatal outcome						
Preterm labour ^b^	3 (50)	8 (33)	3 (18)	23 (79)	11 (73)	48 (48)
Livebirth	5 (83)	22 (73)	15 (68)	23 (82)	11 (73)	77 (76)
Stillbirth	1 (17)	0 (0)	0 (0)	2 (7)	3 (20)	6 (6)
Unknown/open	0 (0)	8 (27)	7 (32)	3 (11)	1 (7)	19 (19)

Numbers are presented as frequency (%) if not otherwise specified. Coronavirus disease 2019 (COVID-19), insufflation of oxygen (O^2^), non-invasive ventilation (NIV), intubation for invasive ventilation (ITN), extracorporeal membrane oxygenation (ECMO), intensive care unit (ICU), standard deviation (SD), interquartile range (IQR). ^a^ If information is taken out of the questionnaire (subgroup analysis): total *n* = 87, monitor N = 6, O^2^ N = 21, NIV N = 22, ITN N = 23, ECMO N = 15. ^b^ Defined as childbirth earlier than 37 weeks of pregnancy; *p* = 0.0004.

## Data Availability

The data presented in this study are not publicly available but available on request from the corresponding author. The data are not publicly available due to privacy and ethical restrictions.
